# Sensing the heat and the host: Virulence determinants of *Histoplasma capsulatum*

**DOI:** 10.1080/21505594.2019.1663596

**Published:** 2019-09-27

**Authors:** Sinem Beyhan, Anita Sil

**Affiliations:** a Department of Infectious Diseases, J. Craig Venter Institute, La Jolla, CA, USA; b Department of Microbiology and Immunology, University of California, San Francisco, CA, USA

**Keywords:** *Histoplasma capsulatum*, dimorphic fungi, fungal morphology, fungal pathogenesis, virulence, Ryp factors

## Abstract

*Histoplasma capsulatum* is a member of a group of fungal pathogens called thermally dimorphic fungi, all of which respond to mammalian body temperature by converting from an environmental mold form into a parasitic host form that causes disease. *Histoplasma* is a primary fungal pathogen, meaning it is able to cause disease in healthy individuals. We are beginning to understand how host temperature is utilized as a key signal to facilitate growth in the parasitic yeast form and promote production of virulence factors. In recent years, multiple regulators of morphology and virulence have been identified in *Histoplasma*. Mutations in these regulators render the pathogen unable to convert to the parasitic yeast form. Additionally, several virulence factors have been characterized for their importance in *in vivo* survival and pathogenesis. These virulence factors and regulators can serve as molecular handles for the development of effective drugs and therapeutics to counter *Histoplasma* infection.

## Introduction

*Histoplasma capsulatum*, a dimorphic fungal pathogen, is the most common cause of fungal respiratory infections in immunocompetent hosts [1,2]. Although it is distributed worldwide, *Histoplasma* is most commonly found in North America, Central America and Africa [1]. It is estimated that over twenty-five thousand individuals acquire life-threatening infections of *Histoplasma* each year with up to a 50% mortality rate [2]. The outcome of infection depends on the dose of infectious particles as well as the immune status of the host; life-threatening respiratory and/or systemic disease (histoplasmosis) occurs most frequently in immunocompromised hosts, or in immunocompetent hosts who have inhaled a large number of infectious particles [1,3]. Host invasion and colonization strategies employed by *Histoplasma* are poorly studied. To date, only a handful of virulence factors have been identified. In this review, we describe previously identified regulators of morphology and virulence, the function of known virulence factors, and our current understanding of host response to *Histoplasma* infections.

### From the environment into the host

*Histoplasma* species are members of a group of fungal pathogens called thermally dimorphic fungi [4]. These fungi respond to mammalian body temperature by converting from an environmental mold form into a parasitic form that causes disease [5]. *Histoplasma* species are found worldwide [6–9].

In the soil, *Histoplasma* grows in a multicellular form comprised of a network of vegetative filaments (hyphae). These hyphae produce asexual spores termed conidia. *Histoplasma* can produce two types of conidia varying in size, therefore named as macroconidia and microconidia. The environmental or clinical significance of these differentially sized conidia is not yet understood. However, it is thought that small size of the microconidia allows them to access alveoli in mammalian lungs. Once inhaled, hyphal fragments and conidia convert to a budding yeast form that expresses virulence genes and causes disease. This morphological transition enables *Histoplasma* to grow to high fungal burden within host immune cells and presumably facilitates the intracellular lifestyle.

### Environmental factors that affect morphological transitions

Mammalian body temperature is critical and sufficient to stimulate the transition from the environmental hyphal form to the pathogenic yeast form in *Histoplasma* [10–12]. Accordingly, in the laboratory, the switch between the environmental and host form is recapitulated by changing the temperature: cells grow in the hyphal form at room temperature, whereas growth at 37ºC is sufficient to trigger growth in the yeast form and expression of virulence factors [13]. Early studies also established that there is an inverse correlation between temperature sensitivity and pathogenicity among *Histoplasma* strains. Specifically, the hypha-to-yeast transition is slower in the temperature-sensitive less virulent Downs strain, and Downs yeast cultures exhibit slower growth at 37ºC in comparison to more virulent strains G184A and G222B [14].

Temperature also serves as a developmental signal for conidia. Even under *in vitro* conditions, conidia can give rise to hyphae at room temperature and to yeast at mammalian body temperature [15]. Upon inhalation by the mammalian host, conidia germinate to give rise to the budding yeast-form [16]. The molecular mechanism of temperature sensing in conidia is not known and the transcriptome of germinating conidia has not been examined. Since hyphae and conidia have distinct transcriptional profiles [15], it is highly likely that the transcriptional changes during the conidia-to-yeast transition will differ from those that occur during the hypha-to-yeast transition. It is also tempting to speculate that conidial transcripts and those transcripts that become abundant during the conidia-to-yeast transition may be enriched in ones that encode virulence factors.

Other than temperature, environmental factors that affect morphological transitions have not been fully explored. One of the earliest studies of *Histoplasma* pointed out that exposure of yeast cells to dark conditions for 48 hours prior to infection can enhance virulence attributes of *Histoplasma* as assessed in a mouse infection model [17]. Furthermore, intracellular cysteine levels and mitochondrial respiration rate were linked to the hyphal-to-yeast transition, and exogenous cysteine promoted the hyphal-to-yeast transition in an early study [18]. Addition of exogenous cAMP was shown to override the yeast program and stimulate hyphal formation in *Histoplasma* even at 37ºC [12]. Similarities and differences between morphologic transitions induced by temperature, light-dark, cysteine, or cAMP have yet-to-be studied at a molecular level in *Histoplasma*. However, the molecular nodes that are important for inducing virulence gene expression in response to temperature are described below.

### Phase-specific gene expression and translation

Gene expression analysis has been frequently used in *Histoplasma* to reveal yeast- and hyphal-phase specific transcripts and to identify genes that are required for yeast-phase growth and virulence. Early studies showed that transcript levels of the genes involved in cell division and structure (α-tubulin (*TUB1*), β-tubulin (*TUB2*) and cyclin-dependent protein kinase (*CDC2*)) change during morphological transitions. *TUB1* and *TUB2* are increased in the hyphal phase whereas *CDC2* transcript levels are increased during yeast-phase growth [19,20]. Additionally, a limited number of genes (e.g. *YPS3* and *CBP1*) that are required for virulence were identified as yeast-phase enriched transcripts by early studies [21–23].

With the advancement of omics technologies, yeast- and hyphal-phase specific gene expression has been profiled in multiple *Histoplasma* strains (G217B, G186AR, H88 and H143) [15,24–28]. These studies confirmed the early findings that previously identified virulence factors (*CBP1, YPS3* and *YPS21*) of *Histoplasma* are all highly-enriched transcripts in the yeast phase. In addition, about 5-10% of genes are found to have yeast-phase specific (YPS) expression patterns and about 5-10% of genes are found to have hyphal-phase specific (HPS) expression patterns in different studies [24–26,28]. Conservation of phase specific gene expression among the *Histoplasma* isolates was analyzed [25,26] and it was shown that about 7.6% of the YPS genes showed lineage-specific expression patterns [25]. Additionally, among genes that are conserved between the four strains, 139 of them have been identified as core YPS genes and 291 have been identified as core HPS genes, meaning that their differential expression is also conserved [26].

In *Histoplasma*, about 2% of the transcripts exhibit phase-dependent variation in 5ʹUTR length [26,27]. The mechanism and significance of this phenomenon has not been explored in *Histoplasma*. However, it is thought that 5ʹUTR length can affect translation rates, thereby introducing an additional level of complexity in regulating protein levels [26]. To this end, Gilmore et al. performed the first ribosome profiling experiments in dimorphic fungi, and showed that a subset of *Histoplasma* transcripts can exhibit altered translational efficiencies, which is in part affected by the variation in 5ʹUTR length [26]. For example, the hyphal-enriched transcript *MS95* has a longer 5ʹUTR sequence in yeast cells vs. hyphae, and displays a lower translational efficiency in yeast cells compared to the hyphal form. Conversely, Ryp2, which a regulator of yeast-phase growth and is required for yeast cell morphology [29], has a longer 5ʹUTR and lower translational efficiency in the hyphal phase. Future studies focusing on the molecular details of the mechanism of transcript length determination and the importance of transcript length in phase-specific gene regulation may shed light on the temperature response in *Histoplasma*.

The only conidial transcriptome study was performed by Inglis et al. using two different *Histoplasma* strains, G217B and G186AR [15]. Comparison of yeast-, hyphal-, and conidial-specific transcripts revealed that about 20-28% of the phase-specific transcripts displayed conserved expression between the two strains. Conidial-enriched transcripts included those involved in stress responses, DNA metabolism and transcriptional regulation [15]. One of the most interesting transcripts that was enriched both in conidia and yeast was *RYP1*, which was originally identified in forward genetic screens to be required for yeast-phase growth [15,28], as discussed below in detail.

### Regulators of yeast phase

Despite the prevalence of *Histoplasma* and its threat to human health, very little is known about the link between cell morphology and expression of virulence factors. Forward genetic screens have been used to identify genes that are important for yeast-phase growth, resulting in the discovery of three key regulators (Ryp1, Ryp2, and Ryp3) [28,29]. Ryp1 is an ortholog of Wor1, which is a regulator of white-to-opaque switching in *Candida albicans* [30,31]. Ryp2 and Ryp3 are Velvet family proteins, which are involved in development in filamentous fungi (reviewed in [32]). Ryp2 and Ryp3 are also required for spore development, spore viability and the conidia-to-yeast transition within macrophages [29]. All three regulators have been shown to have DNA binding activity, and distinct motifs have been defined for Ryp1 and for the Ryp2-3 heterodimer [24,28]. Notably, Ryp1, Ryp2 and Ryp3 form protein complexes [24]. In a comprehensive analysis of Ryp1, Ryp2 and Ryp3 regulons, another transcriptional factor, Ryp4, was identified as a direct target of Ryp1, Ryp2 and Ryp3 [24]. Further analysis showed that Ryp1, Ryp2, Ryp3 and Ryp4 can regulate each other’s expression, and all four factors associate with the upstream regulatory regions of Ryp1, Ryp2, and Ryp4 [24,28,29]. Interestingly, some of the known virulence factors in *Histoplasma* are targets of Ryp1, Ryp2, Ryp3 and Ryp4, confirming the fundamental role of these Ryp factors in regulating virulence traits. Taken together, Ryp factors are master regulators of the yeast-phase transcriptional program, regulating both cell shape changes and virulence gene expression.

Additional regulators of yeast-phase include a histidine kinase, Drk1, which was identified through a forward genetic screen in *Blastomyces dermatitidis*, and was also shown to be required for yeast-phase growth in *Histoplasma* [33]. Furthermore, Velvet protein Vea1 was shown to be required for formation of cleistothecia, which are the mating structures of *Histoplasma* [34]. Vea1 knockdown strains are also defective in hyphal formation at room temperature, and exhibit lower levels of lung and spleen colonization in the mouse model of infection [34]. Future studies will reveal whether Drk1 or Vea1 regulates or acts together with Ryp proteins to control cell morphology and virulence traits in *Histoplasma*.

### The Histoplasma yeast form is an intracellular pathogen of macrophages

During infection, *Histoplasma* is largely an intracellular pathogen of macrophages [13,35,36]. As such, *Histoplasma* has to both avoid innate immune recognition and contend with a variety of anti-microbial mechanisms. Additionally, after robust intracellular replication, *Histoplasma* must exit macrophages before the yeasts can infect the next round of host cells. Here we will discuss how *Histoplasma* counters anti-microbial mechanisms and survives in the host.

## Histoplasma yeast cells deal with Dectin-1

Mammals utilize pattern recognition receptors to detect microbial products, and a number of these receptors recognize elements of fungal cells [37]. Dectin-1 is the host receptor that recognizes β-glucan, a key element of the fungal cell wall. Many *Histoplasma* strains produce α-(1,3)-glucan, a polysaccharide that lies on the outer surface of *Histoplasma* yeast cells (but not hyphae) and shields the underlying β-glucan from recognition by Dectin-1 [38–40]. A number of elegant experiments have shown the key role of α-(1,3)-glucan synthesis in limiting immune recognition and promoting virulence [40,41]. In addition to α-(1,3)-glucan synthesis, elucidation of the *Histoplasma* yeast-phase secretome resulted in the identification of a number of interesting factors [42] including Eng1, a secreted β-(1-3)-glucanase that processes β-glucan in the *Histoplasma* cell wall to limit detection of yeast cells by Dectin-1 [43,44]. Eng1 is required for virulence of *Histoplasma* in wild-type mice but is dispensable in Dectin-1-deficient mice, indicating the key role of Eng1 in modulating β-glucan recognition.

## Countering anti-microbial mechanisms

Recent molecular genetic analysis has highlighted the ability of *Histoplasma* to counter reactive oxygen species (ROS) in the host. Cells of the innate immune system (such as monocytes, polymorphonuclear leukocytes/neutrophils (PMNs), dendritic cells, and macrophages) produce ROS via the NADPH oxidase complex [45]. A number of studies have shown that *Histoplasma* yeasts do not trigger an oxidative burst in resting macrophages [46–48]. In contrast, ROS production is triggered when PMNs encounter *Histoplasma*, and in cytokine-activated macrophages infected with *Histoplasma* [46,48–52]. Analysis of the extracellular proteome of *Histoplasma* yeasts [42] revealed a secreted Cu/Zn-type superoxide dismutase (Sod3) that is associated with the cell wall via a GPI anchor [48]. Whereas the intracellular dismutase Sod1 copes with cytosolic oxidative stress, Sod3 is positioned outside yeast cells to allow them to counter superoxide produced by host macrophages or PMNs. Sod3 is required for survival of *Histoplasma* yeast cells when cultured with PMNs and during infection of activated macrophages [48]. Moreover, Sod3 is required for *Histoplasma* to achieve normal fungal burden and virulence in the mouse model of histoplasmosis. In an elegant experiment to confirm that the role of Sod3 in pathogenesis is to counter ROS produced by phagocytes, Youseff et al. showed that Sod3 is dispensable for virulence in mice that lack a functional phagocyte NADPH-oxidase complex [48].

Superoxide dismutase is not the only enzymatic defense that *Histoplasma* deploys against ROS. Superoxide dismutation results in the production of hydrogen peroxide, which is neutralized enzymatically by catalases [53]. *Histoplasma* expresses three catalase genes: CatA, which is expressed in hyphae, CatB, which is expressed in yeast cells, and CatP, which is expressed in both [54,55]. Holbrook et al. [55] showed that CatB is an extracellular catalase whereas CatP is required for intracellular catalase activity. Furthermore, *Histoplasma* cells that lack both CatB and CatP show substantial defects in survival during co-culture with human PMNs and during infection of activated human macrophages. Similarly, the *catB catP* double mutant has a severe defect in pulmonary fungal burden and dissemination in the mouse model of infection. Therefore it is clear that these two catalases play an important, overlapping role in countering ROS in the context of the host.

## Coping with nutritional limitation in the host

The role of nutritional immunity in restriction of *Histoplasma* infection has been a highlight of a number of studies. It has been known for some time that the cytokine interferon gamma (IFNγ), which is a key player in the adaptive immune response to *Histoplasma*, restricts the amount of iron that is available to the fungus [56]. Murine peritoneal macrophages treated with IFNγ are able to restrict the growth of *Histoplasma*, and growth restriction can be reversed by addition of iron-rich holotransferrin [56]. IFNγ downregulates surface transferrin receptors, suggesting that a major means by which IFNγ inhibits intracellular fungal growth is via iron limitation. *Histoplasma* has a number of iron acquisition pathways that could counter iron restriction in the host [57,58], several of which have been studied on a molecular level. Under conditions of iron limitation, *Histoplasma* induces the production of low molecular weight hydroxamate siderophores [59,60] that facilitate iron uptake without requiring reduction of ferric to ferrous iron. *SID1* encodes L-ornithine-N5- monooxygenase, which catalyzes the first committed step in siderophore biosynthesis. *SID1* is required for optimal colonization of macrophages and mice, suggesting that siderophores play an important role in countering iron restriction during infection [61,62]. Interestingly, *SID1* requirement for *in vivo* colonization is the most obvious at 15 days post-infection [62] perhaps due to IFNγ production by T cells, which restricts iron availability and peaks at 14 days post-infection [63]. Similarly, *VMA1*, which encodes the catalytic subunit A of the *Histoplasma* vacuolar ATPase, is required for *Histoplasma* iron homeostasis as well as virulence in macrophage and mouse models of infection [64]. Specifically, *vma1* mutants cannot grow in iron-limited conditions and this growth defect can be rescued by the addition of siderophore-producing yeasts or siderophores. However, *vma1* mutants are not defective in siderophore production, and the precise role of *VMA1* in iron acquisition is unclear [64]. A second strategy for iron uptake is dependent on extracellular reduction from ferric to ferrous iron [58]. Zarnowski et al. identified Ggt1, a γ-glutamyltransferase enzyme that generates the ferric reductant cysteinylglycine [65]. These authors showed that targeting Ggt1 by RNA interference resulted in decreased virulence in a macrophage model of infection.

More recently, it has become apparent that zinc sequestration is an important strategy used by the host to limit *Histoplasma* growth. Macrophages that are activated with the cytokine GM-CSF are able to restrict *Histoplasma* proliferation [66]. GM-CSF triggers expression of metallothioneins (MTs) in macrophages as well as redistribution of subcellular zinc, thereby reducing zinc availability to intracellular yeasts and activating ROS production. Notably, mutant macrophages lacking metallothioneins MT1 and MT2 are unable to restrict growth of intracellular *Histoplasma* in GM-CSF activated macrophages [67]. On the pathogen side, strains where the *Histoplasma* zinc transporter Zrt2 was depleted by RNA interference show decreased virulence in the mouse model of infection as well as decreased fungal burden at later time points in infection [68].

Genetic screens to identify *Histoplasma* mutants that are deficient in macrophage colonization have also been illustrative regarding the nutritional environment of the macrophage phagosome, as well as the capacity of *Histoplasma* to counter nutritional limitation. This type of forward genetic screen identified the copper transporter Ctr3 as being required for growth in the macrophage phagosome [69]. The Ctr3 transcript is significantly more abundant in yeast over hyphae, and is further induced in culture under copper-limiting conditions. A GFP reporter gene driven by the Ctr3 promoter is induced by yeast cells in the phagosome of macrophages activated with IFNγ, suggesting that these activated macrophages utilize copper restriction to modulate *Histoplasma* intracellular growth. Consistent with this hypothesis is the failure of *ctr3* mutant yeasts to proliferate robustly in IFNγ-activated macrophages. Additionally, when wild-type and mutant strains are mixed in the mouse model of infection, the mutant shows a defect in fitness relative to wild-type, but only later in infection after the onset of the adaptive immune response. These data suggest that Ctr3 is critical for the fungus to counter copper restriction that occurs in the host during infection [69].

In addition to fungal genes that are required to counter iron, zinc, and copper restriction, *Histoplasma* also synthesizes vitamins to counteract the nutrient-poor environment of the phagosome. A forward genetic screen for *Histoplasma* mutants that fail to proliferate in the macrophage phagosome identified a mutant deficient in riboflavin biosynthesis. This mutant, as well as a strain defective in pantothoate biosynthesis, fails to thrive in macrophages and in the mouse model of infection [70]. These studies indicate the importance of *de novo* vitamin synthesis for the virulence of *Histoplasma* yeast cells.

## Histoplasma yeast cells express Cbp1, a protein required for host-cell lysis

Once *Histoplasma* has replicated within macrophages, the yeast cells must exit the macrophage to allow further rounds of infection and intracellular colonization. During infection of macrophages, intracellular *Histoplasma* replication is followed by host-cell death and release of live yeast cells. Cbp1, one of the most highly expressed yeast-specific factors [15,23–28,71], is a small secreted protein that has been implicated in the process of macrophage cell death. Key studies first showed that Cbp1 is a highly abundant protein in yeast cultures supernatants [72]. Interestingly, the *CBP1* gene was the first to be disrupted in *Histoplasma*, thereby representing an important landmark in *Histoplasma* molecular genetics [73]. The *cbp1* mutant is unable to lyse murine macrophage-like cells, and is avirulent in the mouse model of infection [73]. Cbp1 was also identified independently in a genetic screen to identify *Histoplasma* mutants with defects in macrophage lysis [74]. In this study, the authors showed that Cbp1 is dispensable for high intracellular fungal burden in macrophages, and yet the infected host cells do not lyse, implying that Cbp1 promotes macrophage cell death. Death of the macrophage during *Histoplasma* infection is independent of pyroptotic or necroptotis pathways, but is partially dependent on pro-apoptotic factors Bax and Bak. More recently, it has been shown that *Histoplasma* triggers an integrated stress response in infected macrophages that results in induction of the stress-responsive transcription factor CHOP [75]. This stress response is absolutely dependent on Cbp1. Mutant macrophages that lack CHOP or other host components of this stress response pathway are partially resistant to *Histoplasma*-mediated host cell lysis. Moreover, CHOP^−/-^ mutant mice display reduced macrophage apoptosis *in vivo* in response to *Histoplasma* infection, as well as decreased fungal burden and significant resistance to infection [75]. These studies highlight the importance of the Cbp1-dependent host-cell-death pathway in *Histoplasma* infection. Ultimately it is of high interest to define the molecular function of Cbp1 and the precise mechanism by which *Histoplasma* triggers host-cell death.

## Conclusion

Molecular genetics, genomics, and proteomics have been powerful tools to uncover critical regulators and effectors of the pathogenic yeast form of *Histoplasma*. However, many open questions about the transition to the yeast form and *Histoplasma* virulence strategies remain. For example, little is known about how temperature is sensed, how *Histoplasma* blocks phagosome acidification in macrophages [76,77], as well as how *Histoplasma* might employ extracellular vesicles [78,79] to manipulate pathogen and host biology. From uncovering which sensors trigger the development of the parasitic yeast form to uncovering how *Histoplasma* manipulates the cell biology of phagocytes, there are many intriguing mysteries to engage *Histoplasma* researchers for years to come.
